# Postoperative radiotherapy after nipple- or skin-sparing mastectomy: a review of recent institutional and pooled data

**DOI:** 10.3332/ecancer.2018.834

**Published:** 2018-05-11

**Authors:** Jacques Bernier

**Affiliations:** Genolier Cancer Center, Clinique de Genolier, Genolier 1272, Switzerland

**Keywords:** breast cancer, skin-sparing mastectomy, nipple-sparing mastectomy, modified radical mastectomy, postoperative radiotherapy, treatment outcome, late complications

## Abstract

The increasing use of nipple-sparing mastectomy (NSM) and skin-sparing mastectomy (SSM) in the treatment of nonmetastatic breast cancer is justified by considerations linked to their therapeutic index. In selected patients, efficacy results tend to be similar to those observed after radical modified mastectomy and at the same time, subcutaneous mastectomies preserve the patient’s body image. Yet the oncologic safety of the two former surgical approaches is still a matter of debate, also in consideration of the almost complete absence of clinical studies directed to prospective, controlled comparisons between subcutaneous and radical modified mastectomies. In addition, no clear statement—and consequently no consensus—emerges from the rather rare reports addressing the issue of whether or not there exist robust algorithms for guiding decision-making in delivering postoperative radiotherapy after NSM or SSM. The objective of the present review article is to revisit the dataset recently provided by the literature, which might help oncology teams optimise local treatment in this patient population.

## Introduction

Throughout the past two decades, both nipple-sparing mastectomy (NSM) and skin-sparing mastectomy (SSM) have been gaining ground in the surgical management of nonmetastatic breast cancer patients. The main reason for this trend relates to the therapeutic index elicited for NSM and SSM, which was shown to compare favourably with that observed for modified radical mastectomy (MRM). Indeed, the literature suggests that treatment outcomes of NSM, SSM and MRM tend to be similar as regards efficacy endpoints, and at the same time, patients draw a number of advantages from the two former surgical modalities, especially in terms of organ and body image preservation [1, 2–9].

Whether to deliver postoperative radiotherapy (PORT) after NSM or SSM or not is nowadays an issue essentially addressed by multi-disciplinary tumour boards involving not only surgeons and radio-oncologists but also diagnosticians, pathologists and medical oncologists. Often, such a decision-making process turns out to be a challenging task, for no straightforward, prospective comparison between subcutaneous radical mastectomies and MRMs has emerged so far from reports analysing the exact relevance of PORT after NSM and SSM [10, 11].

The objective of this review article is to revisit the messages recently provided by the literature which might help oncology teams optimise local treatment in this patient population.

## Methods

We used search strings on studies addressing the issue of interaction between NSM and SSM and adjuvant radiotherapy, via the following search terms: breast cancer, SSM, NSM, MRM, PORT, treatment outcome and late complications. This review article is essentially based on full articles published since 2000 and retrieved from the PubMed search engine (http://www.ncbi.nlm.nih.gov/pubmed).

## Results

In general, recent reports do not provide any clear statement on the indication of PORT after NSM or SSM. Nevertheless, we can identify, in the recent literature, a number of datasets retrieved from institutional reports, pooled analyses, and an international survey of practices.

### Institutional reports: risks of recurrences after SSM or NSM

In comparison with MRM, NSM and SSM may leave mammary gland in subcutaneous tissues. This observation is corroborated by two datasets, Torresan *et al* [12] reported in 2005. First, residual mammary tissue was still present in about 60% of the surgical specimens from a cohort of 42 patients presenting with stage 0-III disease. Second, there was a direct correlation between the presence of residual breast tissue and skin flap thickness: when it exceeded 5 mm, tumour cells are identified in up to 9.5% of the surgical specimens.

Likewise the involvement of the nipple-areolar complex (NAC) by tumour microfoci was demonstrated by Cense *et al* [13] in nearly 60% of mastectomy samples. The main features related to a higher risk of NAC involvement by tumour are a small distance between the NAC and tumour (<4–5 cm), positive lymph nodes, central tumour location and multicentricity [14]. Nevertheless, local regional relapse rates after NSM are low in patients with early-stage breast cancer [15].

The persistence of mammary tissue after SSM or NSM obviously increases the risk of local failure if tumour cells are present in this residual tissue [16–19]. In this, the more advanced the local disease, the higher the risk of local recurrence. In a retrospective study by Meretoja *et al* [20], the local regional failure rates with a mean follow-up of 70 months were 31 and 5.8% in patients with stage III and stage 0-II, respectively.

After SSM, the most significant risk factors for local recurrence identified in two studies are nodal involvement, tumour size, poorly differentiated carcinomas, and lympho-vascular invasion (LVI) [5, 21]. In other three studies, clinical factors such as tumour size of 4 cm or more, [15, 22, 23] chest wall infiltration [22], and any positive lymph node or >3 positive lymph nodes [15, 22] were significantly linked to higher risk of local regional relapse.

### Institutional reports: impact of PORT on complication rates

In 2007, Meretoja published a report on a cohort of 207 consecutive women who underwent, from 1992 to 2001, SSM and immediate breast reconstruction immediate breast reconstruction (IBR) without any adjuvant radiotherapy [20]. This Finnish study found that with a mean follow-up of 70 months, late complications included native skin flap necrosis (10%), hematoma (10%), infection (3.5%), anastomotic thrombosis (5%) and hernia (3%).

It has been repeatedly substantiated that PORT delivered to reconstructed breast carries a higher risk of complications compared to that of no PORT [24–26]. Before the turn of the century, Hunt *et al* [16] showed that patients treated with immediate, autologous TRAM reconstructions develop fat necrosis and radiation fibrosis in 16% and 11% of cases, respectively. Another study, comparing 39 irradiated implant reconstructed breasts with 338 nonirradiated reconstructions, elicited a higher incidence of capsular contracture in the irradiated group [19].

In a recent report published in 2015, Tang *et al* [27] analysed treatment outcomes in a large population of 982 patients treated from 2007 to 2013 with NSM plus immediate reconstruction [27]. Compared to breasts with no radiotherapy, PORT increased overall complications (10.2 versus 17.5%, *P* = 0.03), nipple loss (0.9 versus 4.1%, P=0.02,), and rates of reconstruction failure (2.2 versus 8.2%, *P* = 0.003). On multivariate regression analysis, PORT (OR, 2.29, *P* = 0.015), age >55 years (OR, 2.03, *P* = 0.04), breast volume ≥800 cm3 (OR, 1.96, *P* = 0.04), smoking (OR, 2.62, *P* = 0.001), and peri-areolar incision (OR, 1.74, *P* = 0.03) were independent risk factors for complications. The authors concluded that despite higher complication rates after PORT, reconstruction failure and nipple/areola necrosis remained infrequent. Moreover, capsule formation can readily be treated with capsulotomy or capsulectomy [28].

### Pooled analysis: the nipple skin-sparing mastectomy model

Out of 17 studies analysed by Janssen *et al* [29] in 2015, only seven provided a detailed description of the radiotherapy modalities. In most studies, the delivered dose was 50 Gy. Radiotherapy indications varied markedly among the institutions. Boneti *et al* [22] and Burdge *et al* [30] delivered this level of dose to the thoracic wall in patients with for tumours >5 cm and/or ≥4 positive axillary nodes. Moyer *et al* [31] delivered PORT when resection margins were ≤1 mm. In another study by Rulli *et al* [32], PORT was delivered only in patients for whom residual mammary tissue had been detected. A significant difference in treatment outcome was found in only one study in a cohort of 216 patients, comparing PORT versus no PORT groups, with local regional failure rate of 8.5% and 28.4%, respectively [23]. The unexpectedly high failure rate observed in the no PORT group is bound to be linked to the inclusion of high-risk patients in this study. Indeed tumours >3 cm were included in the analysis, and 40% of cases were node-positive.

At the European Institute of Oncology in Milan, Petit *et al* [33] performed radiotherapy (RT) exclusively to the NAC as either electron intraoperative radiotherapy or as a single dose of 16 Gy (electron beams) a few days after surgery. The study reported a rate of local regional recurrence of 1.4%, with all recurrences distant from the NAC. Treatment outcomes were not significantly different between the patients receiving ELIOT (*n* = 800) and those receiving delayed PORT (*n* = 201), on 5 days per week [30, 32, 34].

### International survey of practices

Finally, an international survey, conducted by Marta *et al* [35], collected data from 550 physicians (298 radio-oncologists and 252 breast surgeons), mostly practicing in South America (42%), North America, (30%) and Europe (26%). NSM and SSM were performed in more than 15% of newly diagnosed breast cancer patients, by about 30% of the respondents.

Answers to the questionnaire resulted in the identification of nine risk factors, practitioners include into the decision to deliver PORT or not after NSM/SSM and IBR. These factors were: 1) nodal involvement; 2) LVI; 3) grade III carcinomas; 4) triple negative histo-type; 5) young age; 6) positive surgical margins; 7) tumour size; 8) extracapsular extension (ECE) and 9) multi-centric presentation. As expected, radio-oncologists and surgeons did not fully agree on the magnitude of the risk level affecting each of these factors. For instance, the answers from the two professional disciplines most significantly diverged for the first five factors reported here above (with* p* < 0.001 for each of them).

Importantly enough, there was no consensus between surgeons and radio-oncologists with respect to both how to define residual breast tissue after NSM/SSM and the need to assess the presence of residual breast tissue after surgery. In terms of cut-off values for the risk factors, there were also differences in appreciation between the two disciplines: while most radio-oncologists and surgeons tended to consider age below 40 years and tumour exceeding 5 cm in diameter as reasonable cut-offs, there was no full agreement as regards the number of involved nodes: radio-oncologists put the cut-off at one lymph node, surgeons at more than three.

## Discussion

So far, there has been no controlled clinical study that assessed prospectively who will benefit from PORT after NSM or SSM, and who will not. The only messages we can retrieve from recent publications are there based on retrospective analyses of cohorts of patients.

The relevance of historical comparisons is particularly questionable in a surgical field such as subcutaneous mastectomy, especially in consideration of high inter-study heterogeneities in surgical techniques and selection criteria to identify patients amenable to NSM/SSM, with or without PORT.

In a recent analysis of the current role of PORT after NSM, a total of 112,817 patients were isolated from the SEER database [36]. Over the 2006–2010 period, NSM and nonNSM procedures were performed in 470 (0.4%) and 112,347 (99.6%) cases. Patients with 0 nodes/size ≤2 cm, 0 nodes/size 2–5 cm, and unexamined axilla/size ≤2 cm had higher odds of PORT when compared with size- and node-matched mastectomy patients. Multivariate logistic regression showed that NSM patients had higher odds of radiation (OR, 2.01, *P* < 0.001) than mastectomy patients.

Up until recently, the European Institute of Oncology in Milan recommended the use of intraoperative RT to the NAC in this latter patient population [37]. In patients treated with NSM, Petit *et al* [38] indeed showed, in 2012, that risks of recurrences in the breast included high grade tumours, overexpression/amplification of HER2/neu and molecular subtype luminal B. In the nipple areola complex, the corresponding risk factors were age <45 years, absence of oestrogen receptors, high grade tumours, and HER2/neu overexpression and high Ki-67.

Retrospective studies on NAC indicate that 10% to 30% of cases receive PORT [23, 30, 31, 37, 39–46]. They also show large variations in treatment outcomes: local regional recurrence rates vary from 0% to 10%, and failures in the NAC are observed in 0% to 3% of cases. NAC necrosis is reported in 0% to 37% of patients benefiting from NSM.

There is also a grey zone in the literature as regards the specific impact of nodal status on treatment outcome after NSM or SSM. It is therefore legitimate to take into consideration the data retrieved from the Early Breast Cancer Trialists’ Collaborative Group’s meta-analysis [47]. Compared to no adjuvant radiotherapy, PORT delivered after MRM and axillary dissection was found to decrease significantly local regional recurrence rates, whatever the number of positive nodes. This large-scale analysis strongly suggests that all node-positive breast cancer patients treated with SSM or NSM should actually receive PORT.

In the present state of knowledge, the current review strongly suggests that the recommendations by Marta *et al* [35] are in agreement with the messages drawn from both institutional and pooled analyses: following SSM and NSM, PORT has to be delivered to patients with tumour size >5 cm, positive node(s), positive surgical margins or triple negative histo-types. It is also generally accepted that PORT is indicated in patients with thick skin flaps >5 mm, or skin flaps <5 mm with high risk factors such as age <50 years, nodal ECE, lympho-vascular space invasion or multi-centric tumours. Interestingly enough the findings on flap thickness strongly suggest to measure it using magnetic resonance imaging, in order to predict the presence of residual breast tissue status after NSM and SSM.

Finally, this review confirms that, despite the fact that an increase in complications is documented in patients receiving PORT, immediate breast reconstruction using tissue expansion and implant is an acceptable option for women undergoing NSM and SSM for breast cancer [3].

## Conclusions

Whether SSM or NSM is safe in patients to whom no PORT is delivered remains a matter of controversy, especially in the absence of direct, controlled comparisons between patients with or without adjuvant radiotherapy. The literature on PORT after NSM and SSM continues to elicit rather large inconsistencies when treatment efficacy is the main endpoint. In particular, NSM, which spares a small amount of glandular tissue to protect the areola blood supply, raises concerns about the oncologic safety of this type of surgery, especially in high-risk patients. PORT is likely to reduce the risk of local failure in patients treated with subcutaneous mastectomy. Although some reasonable recommendations emerge from large-scale pooled analyses or surveys, the absence of prospective studies still prevents surgeons and radio-oncologists to identify those cases that are bound to draw a clear benefit from PORT after SSM or NSM. This definitely warrants the activation of randomised trials in these clinical settings.

## Conflicts of interest and funding statement

The author has no conflicts of interest and did not receive any funding to publish this review article.
